# The Divine Purpose

**Published:** 1886-10-23

**Authors:** 


### Words of Consolation.

Communications for this column should be addressed, "Chaplain," care of the Editor of The Hospital.

### IV.—The Divine Purpose.

The Divine purpose in suffering may be of three kinds, depending upon the character of the patient. Some are called by God through sickness to a first repentance. Some who have truly repented He chastises, because He loves them, purges them, that they may bring forth more fruit. A third class of sufferers is composed of more advanced Christians who, having learnt to be patient, submissive, and resigned, endure for Christ's sake. These last are martyrs—witnesses for Christ in much tribulation. They need not be exposed to fire, nor to the sword, nor to persecution, yet they are truly martyrs, because through patient endurance in sickness they obtain victories over Satan, and prove themselves heroes of the Cross. People who have not listened to God's voice while in health, and have needed the severer call through the agency of pain, cannot be told at once of the blessings of sickness. They need to be exhorted, rather than comforted; to be convinced of sin before they can realise how their Heavenly Father loves them, and would draw them to Himself. We endeavoured to address these in our article of last week. We would now say a word to sufferers who know that by God's grace they have not repented in vain. You found your Saviour perhaps long ago; and you have not been leading an immoral or unspiritual life. But you have now to learn the meaning of discipline through sickness. You have yet to understand the purging which the Great Vinedresser has ordained, with a view to your bringing forth more fruit. For instance, in health you may never have found yourself very grievously wanting in the virtues of patience and meekness, simply because you were never severely tested. You are sometimes surprised now at the irritability of your temper. You know that you have to put up with much that is hard to bear, and yet you find yourself constantly complaining, or secretly discontented with your lot. You cry with the Psalmist, "Why art thou so vexed, O my soul, and why are thou so disquieted within me?" (Ps. xlii, 14). Yes; but just because you are tried in these ways, does it not seem that God wants to mould your character into a better likeness of Christ? Is He not inviting you to learn of One who is meek and lowly in heart, that *so* you may find rest for your soul, not otherwise? If you think this may be the Divine purpose in your case, study it to the full. See that your present calling is to endure patiently, and you will find real comfort every time you are able to suppress a needless murmur, or to control irritability of temper. Try and make a point of this, and let those around you see that you intend as much. Remember often to offer up a short petition for patience, and you will gradually come to feel that the time you are detained in hospital is very precious for your soul's sake, and for His sake who bore agonies for you without a murmur. "Father, not My will, but Thine be done" (St. Luke xxii, 42).

